# iBarcode.org: web-based molecular biodiversity analysis

**DOI:** 10.1186/1471-2105-10-S6-S14

**Published:** 2009-06-16

**Authors:** Gregory AC Singer, Mehrdad Hajibabaei

**Affiliations:** 1 Biodiversity Institute of Ontario, Department of Integrative Biology, University of Guelph, Guelph, N1G 2W1, Canada

## Abstract

**Background:**

DNA sequences have become a primary source of information in biodiversity analysis. For example, short standardized species-specific genomic regions, DNA barcodes, are being used as a global standard for species identification and biodiversity studies. Most DNA barcodes are being generated by laboratories that have an expertise in DNA sequencing but not in bioinformatics data analysis. Therefore, we have developed a web-based suite of tools to help the DNA barcode researchers analyze their vast datasets.

**Results:**

Our web-based tools, available at , allow the user to manage their barcode datasets, cull out non-unique sequences, identify haplotypes within a species, and examine the within- to between-species divergences. In addition, we provide a number of phylogenetics tools that will allow the user to manipulate phylogenetic trees generated by other popular programs.

**Conclusion:**

The use of a web-based portal for barcode analysis is convenient, especially since the WWW is inherently platform-neutral. Indeed, we have even taken care to ensure that our website is usable from handheld devices such as PDAs and smartphones. Although the current set of tools available at iBarcode.org were developed to meet our own analytic needs, we hope that feedback from users will spark the development of future tools. We also welcome user-built modules that can be incorporated into the iBarcode framework.

## Background

Advancements in DNA sequencing technologies in recent years have resulted in an explosive use of comparative DNA sequence analysis in biological sciences. DNA sequence information has been used in a wide range of applications and for addressing different biological questions from development to evolution and biodiversity. In the early days of molecular biology a handful of sequence analysis software applications existed, several of them have been developed by researchers to address their needs. In last decade or so, development of more robust sequencing platforms, mainly as a result of human and other genome projects, resulted in the introduction of more powerful data analysis packages. Additionally, advancements in computer technologies and applications have been essential for a boom in bioinformatics. With the widespread use of Internet, it soon became an important vehicle for sequence databases such as GenBank. In addition, organizations such as the National Center for Biotechnology Information (NCBI) and the European Bioinformatics Institute (EBI) as well as smaller initiatives and even individual labs started offering some of their services (i.e. search, access to data, analysis and visualization) through web-based portals.

The majority of tools and portals that have been developed for sequence data analysis have been directed towards genome projects data, mainly because of the overwhelming complexity and large size of genomes as compared to sequence of a single gene. Genome browsers and search tools are good examples. This expansion of sequence information from genes to genomes, have also influenced and been applied to biosystematics analysis. For example, the field of phylogenomics [1] argues for the use of genome sequences (either as a whole or several portions) to study evolutionary relationships.

In contrast to this move from genes to genomes, a relatively new approach, DNA barcoding, aims at developing a species-specific sequence library for all eukaryotes, using a small gene region, with the primary mission of enhancing biodiversity analysis [2]. DNA barocding is based on two key principles of minimalism and standardization. While an efficient identification library requires analyzing maximal number of specimens in different taxonomic groups, species-level identification can be achieved by limiting the analysis to small fragments of genomes (i.e. DNA barcodes). A 650 bp fragment of a mitochondrial gene, cytochrome *c *oxidase 1 (CO1, *cox1*) has been proposed as the DNA barcode for animal species [3]. Several studies have demonstrated the effectiveness of this CO1-barcode system in groups such as fishes [4], mammals [5], birds [6] and several arrays of insects [7,8]. While barcoding by using a single gene fragment has proven efficient for most animals tested, it may be necessary to use 2–3 fragments to achieve species-level resolution in other kingdoms of life.

Although DNA barcoding data – sequence information attached to specimens from different species – has similarities to other biosystematics sequence data (i.e. phylogenetic and population genetics data) [9], new analysis tools are required to facilitate efficient use of barcode information in biodiversity studies. One of the most distinctive features of barcode datasets involves relatively large number of barcode sequences (i.e. several thousands) connected to collateral information (i.e. geographic, ecologic). The analysis and visualization of such large datasets have been challenging.

Here we introduce iBarcode.org, a web-based application server that provides various visualization and analysis tools for DNA barcoding data in a user-friendly environment. These tools have mainly been designed to enable the analysis of large barcode-style data sets, although the features can be used for the analysis of other sequence data. iBarcode.org is free and does not require registration.

## Results

The current implementation of iBarcode.org (July 2008) includes a sequence upload and management suite and nine analysis and visualization tools. The sequence upload and management suite enables input, selection, verification, concatenation, and visualization of sequences. The web server provides tools that are divided into three categories. Here we introduce key features of iBarcode.org and provide exemplar cases from barcode data for each analysis and visualization module.

### Sequence analysis

#### a. Haplotype variation

**Figure 1:**
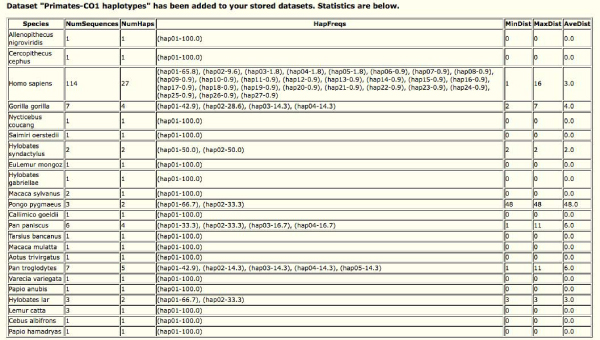
**Screen capture of haplotype variation analysis tool in iBarcode.org**. Basic haplotype statistics for each species is presented in a simple HTML table format easily transferable to word processing or spreadsheet programs.

This tool identifies unique haplotypes for each species and provides statistical information on haplotype frequency and nucleotide variation in a user-friendly table format. A simple measure of number of nucleotide difference between sequences is used to calculate haplotype variation across the sequences. Figure 1 demonstrates the screen capture from output of the haplotype variation tool for a set of primate species (partial data set from Hajibabaei et al. [10]). In addition to this table, a reduced dataset containing unique haplotypes is produced in FASTA format. This dataset is stored for further use in other tools (see below) or for download by the submitter.

#### b. Haplotype map (Barcode-HAPMAP)

**Figure 2:**
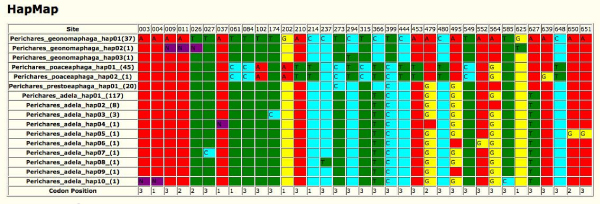
**Barcode-HAPMAP**. An HTML representation of nucleotide characters unique to each haplotype in a set of barcode sequences. The exemplar data is from 4 species of skipper butterflies [12].

This data visualization module provides a graphical view of the nucleotide character variation in a barcode data set. It allows the user to quickly pinpoint nucleotide positions within the barcode sequence that account for barcode variation in a set of species. The tool takes a FASTA alignment of barcode sequences (or the alignment of unique haplotypes created in the Haplotype Analysis tool from a given barcode dataset) as input and highlights variable positions across the barcode sequence in an easy-to-read format. It also shows the nucleotide position for each variable site (counting from 5' to 3') as well as the codon positions they belong to. It is therefore important that the FASTA file of the barcode sequences is in the correct reading frame. This tool works best for focused character-based analysis of a limited number of taxa (i.e. in a species complex or when dealing with cryptic species) as a complement to distance-based methods such as Neighbour-joining analysis [11]. The HTML output format generated by this tool allows robust data transfer to other software packages such as MS-Excel. Figure 2 is an exemplar Barcode-HAPMAP of the unique haplotypes in a set of 4 species of skipper butterflies (Lepidoptera:Hesperiidae) [12].

#### c. Tests of selection at different taxonomic levels

**Figure 3:**
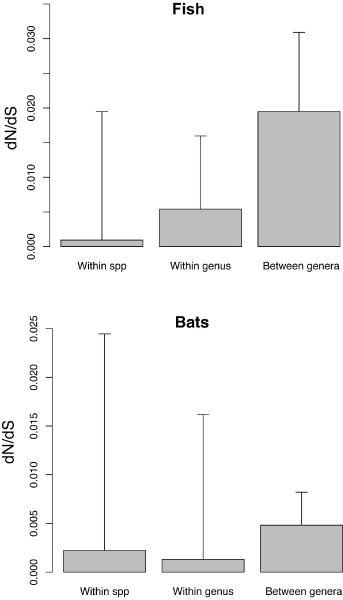
**Test of selection at different taxonomic levels**. Bar graphs representing the ratio of non-synonymous to synonymous nucleotide substitutions (ω) within species, within genus, and between genera for two exemplar data sets of fish [4] and bats [20].

This module uses the popular ratio of non-synonymous to synonymous substitutions (ω) [13] at various taxonomic levels. This ratio has been used for estimating the degrees of selective pressure in molecular biosystematics. The module uses the program yn00 from the PAML package [14,15] to calculate the ratio of non-synonymous to synonymous substitutions (*ω*) for all pairs within a set of aligned sequences. It then calculates the average and standard deviation of *ω *for all sequences pairs that belong to the same species, belong to the same genus, or belong to different genera. A final bar graph depicting these various values is then displayed (Figure 3).

#### d. DNA barcode cloud visualization

**Figure 4:**
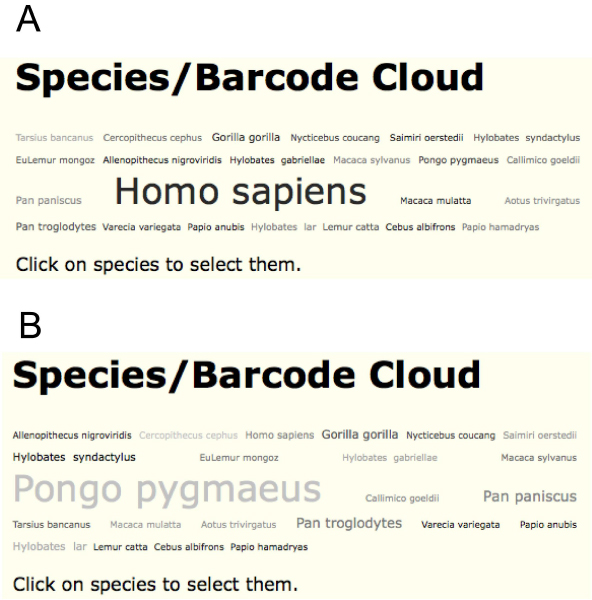
**Species/Barcode cloud graphs tool in iBarcode.org**. A. cloud representation of number of individuals per species for a set of primate CO1-barcodes [10]. B. cloud representation of within species sequence variation for the same primate data set. In each case the font size shows the relative value for each species.

This module takes the popular "word cloud" concept and applies it to number of individuals of each species within a given barcode dataset, producing a visually-appealing means of seeing the relative abundance of species within a dataset. These relative abundances are linearly scaled between font sizes of 50 and 200 points. This feature also provides cloud visualization for sequence divergence within species and haplotype diversity in each species. Each species represented in the cloud visualization output can be selected to create a new subset dataset for further analysis using other tools. Figure 4 provides an example of a barcode cloud for a set of species of primates.

### Genetic distance analysis

#### a. Between- vs. within-species variation graph

DNA barcoding is based on a simple premise: genetic variation between species exceeds that of within species. This tool allows the user to visualize this principle in a given barcode dataset. Specifically, for each species with 3 or more individuals, this tool plots maximum Within Species Divergence (Max-WSD) against minimum Between Species Divergence (Min-BSD) [7]. The input for this tool is a genetic distance matrix (text format) produced either internally (by calculating number of nucleotide differences between and within species) or by common sequence analysis programs such as Mega [16]. Several barcoding studies have used graphs of between- vs. within-species variation. These graphs are considered as one of the standard methods of visualizing barcode data [i.e. [7]], as they allow the user to quickly see outliers that may represent misannotated specimens or sequencing errors.

### Tree analysis

#### a. Organic trees

In Hajibabaei et al. [7], we pioneered a new visually-appealing technique for drawing organic-looking phylogenetic trees. This method maximizes resolution for tips of the tree (i.e. species), which are most important in barcode analysis. The process of building organic trees takes several hours and therefore we have been offering the creation of such trees as an e-mail service.

#### b. Tree collapse

This tool uses bootstrap values in a phylogenetic tree as a benchmark for visualizing statistical support of a given barcode dataset [10]. This is done by collapsing all the branches that are unsupported by a bootstrap cut-off value that is specified by the user. Although short barcode sequences are not strong phylogenetic markers at deep levels, they are excellent for species-level divergences. A high bootstrap cut-off (i.e. 100%) leads to collapsing most of the branches deeper than species-level, but the majority of the species-level branches are kept intact. However, exceptionally closely related species may require longer sequences to gain a very high bootstrap support.

#### c. Tree tip colourization

This visualization tool uses a standard Newick format tree and colourizes the branches leading to individuals of each species (within-species distances) in red and the branches leading to each unique species in blue. It provides a robust method to visually compare different parts of a tree and therefore helps pinpointing exceptional divergence levels or regions of the tree that lack monophyly.

## Server details

iBarcode.org is built on the Python-based web.py application framework [17]. Although most analyses are performed using Python itself, visualization and analysis are accomplished via calls to the statistical language R [18], the graphing package GraphViz [19], and the phylogenetic analysis package PAML [14]. We have intentionally kept the interface light and clean so that it loads quickly over low-bandwidth connections, and so that it is viewable and functional from text-based browsers (such as Lynx) or from small handheld devices (cell phones or PDAs).

In the future, we plan to have an application programming interface (API) for our tools, allowing other developers to integrate our analyses into their own tools.

## Conclusion

Similarly to several other branches of biology, biodiversity science has increasingly been relying on DNA sequence information. DNA barcoding, as a new global initiative for biodiversity analysis, demands specialized bioinformatics tools and applications. iBarcode.org is a web-based application server developed for visualization and analysis of DNA barcode data. The suite of simple but highly customized tools in iBarcode.org allows the analysis and visualization of barcode data at sequence, genetic distance, and phylogenetic tree levels. Several of these applications have already contributed to barcode publications. iBarcode.org provides a web2.0 environment for developing and sharing tools for barcode data and sets the stage for a new wave of community driven bioinformatics applications.

## Competing interests

The authors declare that they have no competing interests.

## Authors' contributions

GAS designed the server and developed various tools and applications and edited the manuscript. MH conceived the idea, designed several tools and applications and wrote the manuscript.

## References

[B1] Murphy WJ, Pevzner PA, O'Brien SJ (2004). Mammalian phylogenomics comes of age. Trends Genet.

[B2] Marshall E (2005). Taxonomy. Will DNA bar codes breathe life into classification?. Science.

[B3] Hebert PDN, Cywinska A, Ball SL, deWaard JR (2003). Biological identifications through DNA barcodes. Proc Biol Sci.

[B4] Ward RD, Zemlak TS, Innes BH, Last PR, Hebert PDN (2005). DNA barcoding Australia's fish species. Philos Trans R Soc Lond B Biol Sci.

[B5] Hajibabaei M, Singer GA, Clare EL, Hebert PDN (2007). Design and applicability of DNA arrays and DNA barcodes in biodiversity monitoring. BMC Biol.

[B6] Hebert PDN, Stoeckle MY, Zemlak TS, Francis CM (2004). Identification of birds through DNA barcodes. PLoS Biol.

[B7] Hajibabaei M, Janzen DH, Burns JM, Hallwachs W, Hebert PDN (2006). DNA barcodes distinguish species of tropical Lepidoptera. Proc Natl Acad Sci USA.

[B8] Smith MA, Woodley NE, Janzen DH, Hallwachs W, Hebert PDN (2006). DNA barcodes reveal cryptic host-specificity within the presumed polyphagous members of a genus of parasitoid flies (Diptera: Tachinidae). Proc Natl Acad Sci USA.

[B9] Hajibabaei M, Singer GAC, Hebert PDN, Hickey DA (2007). DNA barcoding: how it complements taxonomy, molecular phylogenetics and population genetics. Trends Genet.

[B10] Hajibabaei M, Singer GAC, Hickey DA (2006). Benchmarking DNA barcodes: an assessment using available primate sequences. Genome.

[B11] Saitou N, Nei M (1987). The neighbor-joining method: a new method for reconstructing phylogenetic trees. Mol Biol Evol.

[B12] Burns JM, Janzen DH, Hajibabaei M, Hallwachs W, Hebert PDN (2008). DNA barcodes and cryptic species of skipper butterflies in the genus Perichares in Area de Conservacion Guanacaste, Costa Rica. Proc Natl Acad Sci USA.

[B13] McDonald JH, Kreitman M (1991). Adaptive protein evolution at the Adh locus in Drosophila. Nature.

[B14] Yang Z (1997). PAML: a program package for phylogenetic analysis by maximum likelihood. Comput Appl Biosci.

[B15] Yang Z, Nielsen R (2000). Estimating synonymous and nonsynonymous substitution rates under realistic evolutionary models. Mol Biol Evol.

[B16] Kumar S, Tamura K, Nei M (2004). MEGA3: Integrated software for Molecular Evolutionary Genetics Analysis and sequence alignment. Brief Bioinform.

[B17] web.py. http://webpy.org.

[B18] R. http://www.r-project.org.

[B19] GraphViz. http://www.graphviz.org.

[B20] Clare EL, Lim BK, Engstrom MD, Eger JL, Hebert PDN (2007). DNA barcoding of Neotropical bats: species identification and discovery within Guyana. Mol Ecol Notes.

